# Why Didn't the Sifaka Cross the Road? Divergence of *Propithecus edwardsi* Gut Microbiomes Across Geographic Barriers in Ranomafana National Park, Madagascar

**DOI:** 10.1002/ajp.23732

**Published:** 2025-02-04

**Authors:** Mariah E. Donohue, Alicia Lamb, Abigail E. Absangba, Eliette Noromalala, David R. Weisenbeck, Rebecca M. Stumpf, Patricia C. Wright

**Affiliations:** ^1^ Department of Biology University of Kentucky Lexington Kentucky USA; ^2^ Department of Biological Sciences Binghamton University Binghamton New York USA; ^3^ Department of Ecology and Evolution Stony Brook University Stony Brook New York USA; ^4^ The Wild Center Tupper Lake New York USA; ^5^ Department of Anthropology University of Illinois Urbana‐Champaign Urbana Illinois USA; ^6^ Department of Anthropology New York University New York New York USA; ^7^ Anthropobiologie et Développement Durable Université Antananarivo Antananarivo Maryland USA; ^8^ Department of Anthropology The University of Texas at Austin Austin Texas USA; ^9^ Centre ValBio Research Station Fianarantsoa Maryland USA; ^10^ Department of Anthropology Stony Brook University Stony Brook New York USA

**Keywords:** allopatry, biogeography, diversity, geographic barrier, habitat disturbance, lemur, Madagascar, microbiome, primate

## Abstract

This study uses a biogeographic framework to identify patterns of gut microbiome divergence in an endangered lemur species endemic to Madagascar's southeastern rainforests, the Milne‐Edwards's sifaka (*Propithecus edwardsi)*. Specifically, we tested the effects of (1) geographic barriers, (2) habitat disturbance, and (3) geographic distance on gut microbiome alpha and beta diversity. We selected 10 social groups from 4 sites in Ranomafana National Park with varied histories of selective logging. Sites were spaced between 4 and 17 km apart falling on either side of two parallel barriers to animal movement: the Namorona River and the RN25 highway. Using 16S rRNA metabarcoding, we found the greatest beta diversity differentiation to occur between social groups, with significant divisions on opposite sides of geographic barriers (road/river). Habitat disturbance had the most significant effect on alpha diversity, though, contrary to many other studies, disturbance was associated with higher microbial species richness. Without biomedical context, it is unclear whether microbiome differences observed herein are neutral, adaptive, or maladaptive. However, microbiome divergence associated with the road/river may be a symptom of reduced host gene flow, warranting further investigation and perhaps conservation action (e.g., construction of wildlife bridges). Finally, this work demonstrates that significant microbiome variation can accrue over small sampling areas, lending new insight into host‐microbe‐environmental interactions.

## Summary

- Significant gut microbiome divergence accrued on either side of a geographic barrier to *Propithecus edwardsi* movement (a parallel river and highway).
- *P. edwardsi* populations in sites with more recent habitat disturbance had higher gut microbiome alpha diversity.
- Geographic distance did not impact gut microbiome alpha or beta diversity.

## 1 Introduction

The gut microbiome offers key insight into the ecology and evolution of Primate hosts. This is particularly true of gut bacteria, for which community structure (i.e., diversity and composition) is inextricably linked with host diet, habitat, and phylogeny (Donohue et al. 2023). These tight correlations reflect millions of years of co‐evolution and ecological filtering (Amato et al. 2019), driven by the important roles bacteria play in host health (Peixoto, Harkins, and Nelson 2021), fitness (Brooks et al. 2016), and survival (Worsley et al. 2021).

Comparing gut microbiome variation in different ecological contexts helps us to understand how species have adapted to their environments and can inform conservation action (Stumpf et al. 2016; West et al. 2019). In Primates, many previous studies have reported gut microbial variation associated with habitat disturbance, ecosystem characteristics, and feeding behavior (e.g., Amato et al. 2013; Barelli et al. 2015; Gomez et al. 2015; Trosvik et al. 2018; Umanets et al. 2018; Donohue et al. 2019; Greene, Bornbusch, et al. 2019; Greene, Clayton, et al. 2019). This growing body of literature highlights the gut microbiome as an interface with the external environment that can trigger a range of physiological outcomes for Primate hosts, from increased parasite load (e.g., de Winter et al. 2020) to adaptations for dietary specialization (e.g., Greene et al. 2020). The gut microbiome is also a reflection of familial relationships and social dynamics. Infants initially acquire microbes through genetic and maternal factors, as well as environmental exposure (McKenney, Rodrigo, and Yoder 2015). Affiliative interactions, such as grooming, lead to further microbial exchange—especially for lemurs, which use their toothcomb to groom, consequently acquiring microbes present on group members’ hair and anogenital regions (Clough, Heistermann, and Kappeler 2010). Indeed, many lemur studies have found group membership and social networks to be important predictors of microbiome composition and diversity (e.g., Perofsky, Lewis, and Meyers 2019; Raulo et al. 2017; Murillo et al. 2022).

Due to the combined homogenizing effects of social interactions plus shared environment, diet, and genetic relatedness, populations living in sympatry tend to share more similar microbiomes (e.g., Greene et al. 2019a; Rudolph et al. 2022). An important next step in our understanding of host‐microbe evolution is mapping how the microbiome diffuses across a dynamic landscape. Studies focused on population genomics and speciation have shown that barriers to animal movement, such as roads and rivers, are known to catalyze phenotypic and genotypic divergence between populations (e.g., Brunke et al. 2019; Maigret, Cox, and Weisrock 2020). In allopatry, even with some degree of migration and gene flow, intraspecific differences accumulate steadily over time as a consequence of ecological adaptation and drift (Rundle and Nosil 2005). Thus, geographic barriers act as an engine of biodiversity for many taxa. Whether this is the case for the gut microbiome remains largely untested, particularly within the Primate order.

Milne‐Edwards's sifakas *(Propithecus edwardsi)* of Ranomafana National Park (RNP) offer a compelling system for studying the gut microbiome in a biogeographical context. The subject of long‐term research for over 35 years, many facets of *P. edwardsi* behavior, ecology, movement, and genetics have already been described (e.g., Wright 1995, 1998; Morelli et al. 2009; Pochron et al. 2003; Arrigo‐Nelson 2006; Matos, Fernandes, and Wright 2022). *P. edwardsi* tends to form small groups, ranging from two to nine individuals (Wright 1998). Groups demonstrate considerable variation in composition, with longitudinal studies documenting relatively equal proportions of multimale/multifemale groups, harem males, and polyandrous females (Pochron et al. 2004). Both males and females disperse from their natal groups at equal rates (Morelli 2008). Intergroup contact is rare, as group home ranges do not overlap and home range fidelity is high (Gerber et al. 2012).

Much work to date has leveraged Ranomafana's heterogenous history of anthropogenic disturbance to understand its effects on sifakas, as sections near the RN25 highway were selectively logged in the 1980s and continue to recover today. Home range sizes vary across populations and tend to correlate with habitat disturbance; those in previously logged forests had an average home range of 24 hectares compared with 46 hectares in pristine forests (Gerber et al. 2012). Across sites, the *P. edwardsi* diet is primarily composed of leaves, fruit, and seeds (Matos, Fernandes, and Wright 2022), with seasonal ingestion of Hemiptera nymphs (Wright, pers. obs). Recent fecal metabarcoding evidence suggests they may also consume Cicadellidae (leafhoppers) and Formicidae (ants) (Rowe et al. 2021). To date, no study has systematically examined the *P. edwardsi* gut microbiome. Studies of congenerics have shown hindgut fermentation, and gut microbiota is essential for digestion of fibrous leaves (Greene et al. 2023). Relatedly, the intestinal anatomy of *Propithecus* includes a very long small intestine for their body weight and a 24‐h transit time of food through the gut (J. L. Campbell et al. 2000).

In this study, we examined gut microbiome variation among 10 *P. edwardsi* groups spanning four sampling sites in RNP. We were specifically interested in comparing the relative effects of (1) geographic barriers, (2) habitat disturbance, and (3) geographic distance on microbial alpha and beta diversity. We also tested the effects of group identity, sex, and life history stage, to provide a more comprehensive view of gut microbiome variation. In combination, this study offers new insights into how gut microbiomes diverge between closely related populations and over small geographic areas.

## 2 Methods

### 2.1 Ethics Statement

The methods used for noninvasive fecal collections were approved by the Stony Brook University IACUC committee (IACUC #2016‐2255‐USDA‐NF) and Madagascar National Parks. This research adheres to the American Society of Primatologists Principles for the Ethical Treatment of Non‐Human Primates.

### 2.2 Site Description

**Figure 1:**
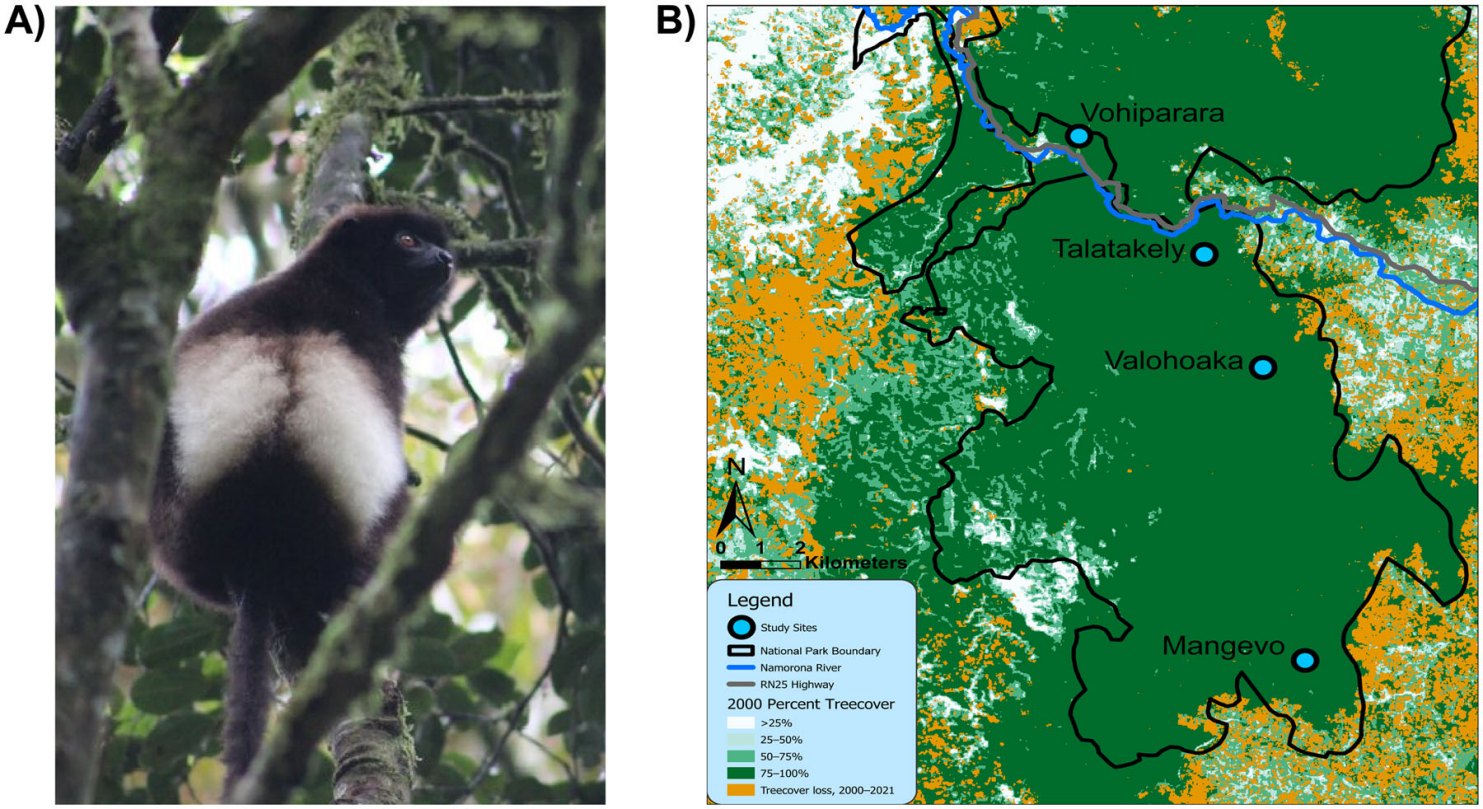
(A) Photograph of a *Propithecus edwardsi* in Talatakely, RNP. Photo credit: Mariah Donohue. (B) Map of study sites and locations of Namorona River and RN25 Highway. All work was completed in Ranomafana National Park, which was mapped using data from UNEP‐WCMC and IUCN (2023). Basemap is drawn from % tree cover in 2000 (shades of green) overlain with tree cover loss from 2000 to 2016 (Hansen et al. 2013; updated 2021).

Fieldwork was conducted in RNP, a 43,500 ha montane rainforest in southeastern Madagascar. Sampling occurred in four sites, each inhabited by multiple resident *P. edwardsi* groups: Vohiparara, Talatakely, Valohoaka, and Mangevo (Figure 1). At each site, sampling occurred within an area of approximately 300 hectares. Three sites (Talatakely, Valohoaka, and Mangevo) are encompassed within Ranomafana's continuous southern block, with no major barriers to lemur movement, while Vohiparara is separated by the Namorona River and the RN25 highway. The sites furthest apart, Vohiparara and Mangevo, are separated by 16.31 km (Table 1).

**Table 1:** Pairwise straight‐line distances between sites, measured in kilometers.

Site	Vohiparara	Talatakely	Valohoaka	Mangevo
Vohiparara	x	4.67	8.19	16.31
Talatakely		x	3.62	12.10
Valohoaka			x	8.6
Mangevo				x

Despite falling on opposite sides of the river, the two northernmost sites (Vohiparara and Talatakely) share many important ecological characteristics. Both are high‐elevation sites (Vohiparara: 1075–1275 m; Talatakely: 900–1100 m) with histories of selective logging before the park was established in 1991, creating a modern‐day mixture of primary and secondary forests (Wright et al. 2011). These sites are also the two most popular eco‐tourist destinations in RNP. Due to habitat disturbance, a major *Propithecus* fruit competitor, the black and white ruffed lemur, *Varecia variegata*, was absent from both sites at the time of sampling (dry season 2016), though as of 2020, *V. variegata* has returned to Talatakely (Donohue, pers. obs).

The two southernmost sites (Valohoaka and Mangevo) are also quite similar ecologically. Valohoaka has an elevation range closer to Talatakely than Mangevo (900–1100 m; Mangevo: 565–1065 m), but both are primary forests with no histories of systematic habitat disturbance. These sites are seldom visited by ecotourists, though they contain long‐term research camps with consistent human presence. *V. variegata* is abundant in both Valohoaka and Mangevo. For simplicity, hereafter, we refer to Vohiparara and Talatakely as “disturbed” forests and Valohoaka and Mangevo as “pristine” forests.

### 2.3 Sample Collection

We collected 118 fecal samples from 41 wild *P. edwardsi* individuals belonging to 10 different groups without overlapping home ranges (Table 2; Table S1). All but three groups (VO3, M1, and M2) were the subject of long‐term research in Ranomafana. All sampling occurred in the dry season, from June to August 2016. The field team started sampling at the focal group's sleep tree(s) each day, where morning fecal samples were collected for each group member (excluding infants) when possible. If any individuals were missed during the morning fecal collection, samples were collected later in the day. Individuals were sampled between one and five times, with most sampled an average of three times. Individuals were identified by previously placed collars and tags. Samples were collected using sterilized tweezers, extracting ~6–10 g of fecal aliquots from the center of the deposit to minimize contact with environmental contaminants on the forest floor. Feces were preserved in 2 mL cryotubes of 96% ethanol, shaken immediately for 30 s to maintain sample integrity, and stored in a −80°C freezer until shipment to the Stumpf Lab for processing and sequencing at the Carver Biotechnology Center at the University of Illinois at Urbana‐Champaign.

**Table 2:** Sampling across sites, groups, and individuals.

Site	Group name	Total # individuals in group	# Samples per individual	# Samples
Vohiparara	VO1	7	1 | 4 | 3	19
—	VO2	5	1 | 4 | 3.2	16
—	VO3	N/A	N/A	1
—	VO4	6	1 | 5 | 3	18
Talatakely	T1	3	3 | 6 | 5	15
—	T4	5	1 | 4 | 2.8	16
Valohoaka	VA1	5	1 | 4 | 2.8	14
—	VA2	4	3 | 4 | 3.5	13
Mangevo	M1	4	1 | 2 | 1.25	5
—	M2	N/A	N/A	1

*Note:* The three numbers in the column “# Samples per individual” show the minimum, maximum, and average number of samples per individual in each group. “N/A” indicates that details about the group are unknown and samples were collected opportunistically.

### 2.4 Data Generation

DNA was extracted using the QIAamp Powerfecal DNA kit (Qiagen Inc.) following the manufacturer's protocol after washing samples in 500 μL 1X PBS to remove the ethanol preservative. We then PCR amplified the V3–V5 region of the prokaryotic 16S rRNA gene using: 2.00 μL genomic DNA, 12.62 μL molecular‐grade water, 2.00 μL 10x PCR buffer, 1.20 μL 50 mM MgCl2, 0.80 μL 10 mM dNTPs, 0.60 mL of each 10 μM primer, and 0.18 μL Platinum Taq DNA Polymerase (Life Technologies). The PCR was performed using the following primer set: 8F (5′‐AGA GTT TGA TCC TGG CTC AG‐3′) and 1492R (5′‐GGT TAC CTT GTT ACG ACT T‐3′). PCR product was confirmed with agarose electrophoresis and quantitative DNA ladder (Hyperladder I, Bioline USA, Boston, MA). DNA samples were loaded onto 1% agarose gel, stained with ethidium bromide, and visualized under UV light.

PCR samples underwent amplicon library synthesis on the Fluidigm Access Array™ System at the Roy J. Carver Biotechnology Center at the University of Illinois at Urbana‐Champaign. Pooled amplicons were then sequenced via Illumina MiSeq.

### 2.5 Bioinformatics

Raw sequences were processed using QIIME2 (Bolyen et al. 2019). We filtered, denoised, and removed chimeras from forward reads using the DADA2 pipeline (Callahan et al. 2016). After these initial quality controls, we retained 1,771,862 sequences (8863–50,755 per sample; mean: 14,890) from 118 samples. Then, we assigned taxonomy based on 99% similarity clustering after consensus blasting reference reads to the SILVA database (SILVA_132 release; Quast et al. 2012). Next, we removed amplicon sequence variants (ASVs) that were detected less than 10 times in the data set and/or could not be assigned to a prokaryotic organism. For downstream diversity analyses, we built rooted microbial phylogenetic trees using the “align‐to‐tree‐mafft‐fasttree” pipeline. Finally, we rarefied our data set at 8863 sequences, the smallest library size in the data set, to maximize sample retention and microbial species diversity.

### 2.6 Data Analysis

The primary goal of our analyses was to elucidate the geographic and demographic factors driving gut microbiome variation in *P. edwardsi* of RNP. Before performing analyses, we first had to account for statistical nonindependence arising from repeated sampling of individuals. To do this, we used two approaches: (1) we calculated the average frequency of each ASV across samples for each given individual using the mean ceiling method (as in Donohue et al. 2022) and (2) we randomly selected one sample per individual and replicated each analysis 10 times.

Our initial set of analyses described taxonomic variation in bacterial taxa across samples. For these analyses, we only present results related to region because other geographic study variables are confounded with locality, making the results repetitive. First, we used count data to determine the relative abundance of bacterial phyla across geographic regions, and then visualized these results with a bar plot using ggplot2 (Wickham 2009). We next sough to identify ASVs whose relative abundance shifted across regions. To do this, we used the analysis of composition of microbiomes (ANCOM) method, which was developed to circumvent traditional issues in analyses of microbial relative abundance such as false positives and biases from underlying data structure (Mandal et al. 2015). ANCOM assesses significance using W‐statistics, a ratio comparing the number of times a given microbial taxon was differentially abundant in relation to all microbial taxa in the data set. Generally, higher W‐values indicate greater differential abundance.

Our next set of analyses tested differences in community diversity using alpha and beta diversity metrics. Alpha diversity measures species richness, while beta diversity measures similarity (or dissimilarity) in community composition of species between samples. To calculate each metric, we used the QIIME2 “diversity core‐metrics‐phylogenetic” command. We then ran Adonis regression models to extrapolate the relative effects of geography and demography on beta diversity (response variable) using the formula “orientation/disturbance/region/group + sex + age” (independent variables), in which “orientation” denotes north or south of the road/river and “region” refers to one of the four sampling sites (Vohiparara, Talatakely, Valohoaka, or Mangevo). Adonis tests were run with 999 permutations using additive sums of squares. For alpha diversity, we ran ANOVA tests in R using the same formula as stated for Adonis, but with alpha diversity metrics as the response variables.

Finally, to determine whether populations in closer geographic proximity shared more similar gut microbiomes, we also conducted pairwise PERMANOVA comparisons for the independent variable *sampling site*. This analysis yields a *q*‐value, which is a *p* value adjusted for false discovery rate to control for multiple comparisons.

## 3 Results

**Figure 2:**
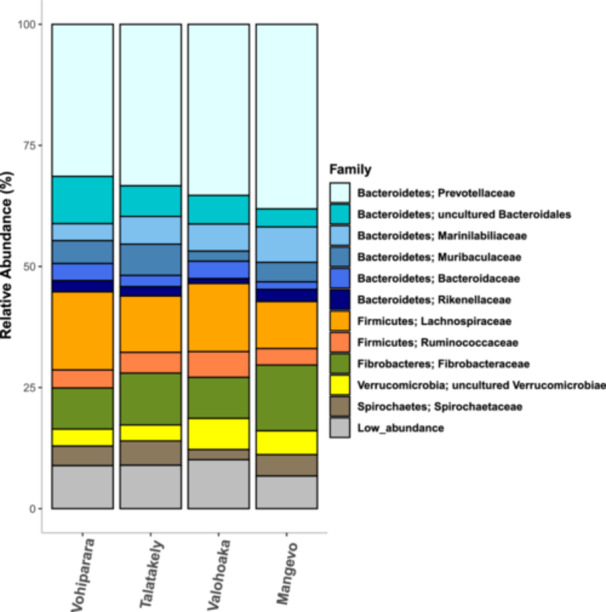
Relative abundance of bacteria families, grouped by sampling site.

In total, we detected 2573 unique ASVs after rarefaction. These ASVs comprised 9 phyla, 25 classes, 31 orders, 65 families, and 95 genera of bacteria. Across samples, we found that about 35% of ASVs belonged to the bacterial family Prevotellaceae within the Bacteroidetes phylum. Also of high abundance were the families Lachnospiraceae (phylum: Firmicutes) (13%), Fibrobacteraceae (phylum: Fibrobacteres) (10%), Marinilabiliaceae (phylum: Bacteroidetes) (6%), Muribaculaceae (phylum: Bacteroidetes) (4%), and an uncultured group of Bacteroidetes (6%) (Figure 2).

ANCOM identified 14 microbial genera with differential abundances across sites (Table 3). We hesitate to interpret these results in the context of other ecological study variables, as, for example, the same microbes associated with Talatakely are also associated with “south of road/river” and “disturbed habitat.” Nonetheless, some interesting patterns arose. In particular, eight genera were more abundant in disturbed sites: (1) unidentified genus in Bacteroidaceae, (2) unidentified genus in Coriobacteriaceae, (3) *Coprobacillus*, (4) unidentified genus in Marinilabiaceae, (5) *Bacteroide*s, (6) unidentified genus in S24‐7, (7) *Sphaerochaeta*, and (8) *Anaerostipes*. Three of these genera followed a pattern of decreasing abundance from north to south (unidentified genus in Bacteroidaceae, unidentified genus in Coriobacteriaceae, and *Sphaerochaeta*), suggesting biogeographic causation (Table 3).

**Table 3:** Differential abundance of prokaryotic taxa across sampling sites using ANCOM.

Differentially abundant genera	Pattern	*W*‐value
Family: Bacteroidaceae	Vohiparara > Talatakely > Valohoaka > Mangevo	86^a^, ^b^
Family: Coriobacteriaceae	Vohiparara > Talatakely > Valohoaka > Mangevo	85^a^, ^b^
Family: Enterobacteriaceae	Talatakely > Valohoaka > Vohiparara; absent in Mangevo	85
Family: Enterobacteriaceae	Valohoaka > Talatakely; absent in Vohiparara and Mangevo	84
Family: Synergistales	Valohoaka > Vohiparara; absent in Mangevo and Talatakely	83
Succinivibrio	Valohoaka > Talatakely > Vohiparara > Mangevo	83
Coprobacillus	Vohiparara > Talatakely > Mangevo > Valohoaka	80^a^
Family: Marinilabiaceae	Talatakely > Vohiparara; absent in Vohiparara and Mangevo	76^a^
Bacteroides	Talatakely > Vohiparara > Valohoaka > Mangevo	71^a^
Prevotella	Valohoaka > Talatakely > Vohiparara > Mangevo	71
Family: S24‐7	Talatakely > Vohiparara > Valohoaka > Mangevo	71^a^
Family: YS2	Valohoaka > Talatakely > Vohiparara > Mangevo	70
Sphaerochaeta	Vohiparara > Talatakely > Valohoaka > Mangevo	68^a^, ^b^
Anaerostipes	Vohiparara > Talatakely > Mangevo > Valohoaka	65^a^

*Note:* In the “differentially abundant genera” column, microbial genera were classified to the lowest possible taxonomic identity. If the genus was unknown, it was listed by family. So, the first line of the “pattern” column should be interpreted as follows: an unidentifiable genus in the Bacteroidaceae family was most abundant in Vohiparara, followed by Talatakely, Valohoaka, and Mangevo, respectively.

^a^ Potential associations with the study variable history of habitat disturbance.

^b^ Results follow a biogeographic pattern of decreasing abundance from north to south.

**Figure 3:**
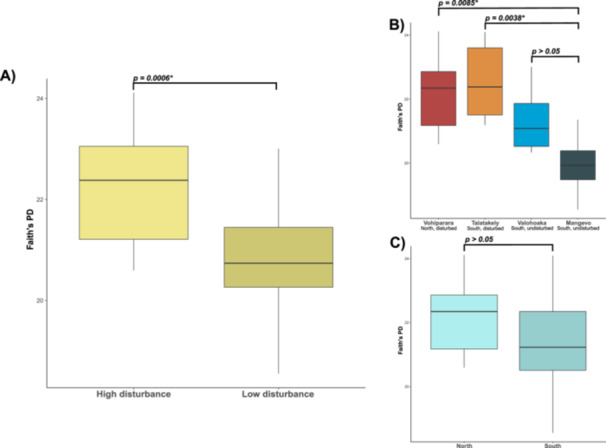
Boxplots of gut microbiome alpha diversity using the mean‐ceiling data set, measured with Faith's PD. Error bars show standard deviation of the mean. (A) Habitat disturbance: Samples from sites with more extensive histories of logging and eco‐tourism harbored greater alpha diversity. (B) Sampling site: Mangevo samples exhibited lower microbiome alpha diversity than Vohiparara and Talatakely. All other pairwise comparisons were not significant (*p* > 0.05). (C) Orientation to the road/river: There were no significant differences in alpha diversity on either site of the road/river.

In the mean‐ceiling data set, habitat disturbance had the largest effect on alpha diversity across all three metrics, with higher diversity in more disturbed sites (Figure 3; Table 4). The relative importance of factors with lower effects varied across metrics. For both faith's PD and observed OTUs, sampling site had the second largest effect. However, social groups had the second largest effect using the Shannon's Index. Social group was significant across metrics, while the effects of orientation to the road/river, life history stage, and sex were largely insignificant. Results in the random replicate datasets were quite different from the mean‐ceiling data set. Habitat disturbance was significant and had the highest effect size in 9 of 30 tests (Table S2). No other variables were significant, with the exceptions of sex (1 of 30 tests) and social group (1 of 30 tests).

**Table 4:** The relative effects of demographic and ecological variables on gut microbiome alpha diversity.

Alpha diversity metric	Variable	*F*‐value	*p*
Shannon's Index	Social group	8.135	0.0002*
	Orientation to the river	5.945	0.0254*
	Site	5.536	0.0302*
	History of disturbance	36.19	< 0.0001*
	Life history stage	1.126	0.3461
	Sex	4.914	0.040*
Faith's PD	Social group	2.695	0.048*
	Orientation to the river	4.144	0.0568
	Site	7.560	0.0132*
	History of disturbance	17.348	0.0006*
	Life history stage	1.073	0.3630
	Sex	0.284	0.6004
Observed OTUs	Social group	8.783	0.0001*
	Orientation to the river	3.715	0.0699
	Site	19.259	0.0004*
	History of disturbance	43.299	< 0.0001*
	Life history stage	0.992	0.3901
	Sex	2.068	0.1676

*Note:* Results of ANOVA tests using the mean‐ceiling dataset. Habitat disturbance drove the greatest gut microbiome variation across alpha diversity metrics.

* *p* < 0.05.

Mean‐ceiling Adonis tests showed social group had the greatest effect size on gut microbiome beta diversity variation across metrics (average *R* ^2^ = 0.23; Table 5), followed by orientation to road/river (average *R* ^2^ = 0.18)*.* Site and habitat disturbance had similar effect sizes (both average *R* ^2^ = 0.09), while life history stage and sex had no significant effects (Table 4). These results were largely mirrored in the random replicate datasets, which showed social group had the greatest effect size in 39 of 40 tests (Table S3). Orientation to the road/river had the greatest effect size in 1 of 40 tests, and the second greatest effect in 33 others. All tests where orientation to the road/river was ranked third or lower were performed using the Weighted UniFrac metric.

**Table 5:** The relative effects of demographic and ecological variables on gut microbiome beta diversity.

Beta diversity metric	Study variable	*R* ^2^	*p*
Bray–Curtis	Social group	0.27	0.001*
	Orientation to the river	0.26	0.001*
	Site	0.09	0.002*
	History of disturbance	0.13	0.002*
	Life history stage	0.05	0.08
	Sex	0.02	0.215
Jaccard	Social group	0.24	0.001*
	Orientation to the river	0.13	0.001*
	Site	0.07	0.001*
	History of disturbance	0.07	0.001*
	Life history stage	0.06	0.054
	Sex	0.03	0.163
Unweighted UniFrac	Social group	0.21	0.006*
	Orientation to the river	0.17	0.001*
	Site	0.08	0.001*
	History of disturbance	0.08	0.001*
	Life history stage	0.05	0.05
	Sex	0.02	0.75
Weighted UniFrac	Social group	0.33	0.001*
	Orientation to the river	0.16	0.001*
	Site	0.11	0.001*
	History of disturbance	0.08	0.003*
	Life history stage	0.03	0.50
	Sex	0.009	0.65

*Note:* Results of Adonis tests of beta diversity using the mean‐ceiling approach. Social group drove the greatest gut microbiome variation across beta diversity metrics.

* Significant *p* value (< 0.05).

Pairwise PERMANOVA tests showed geographic distance did not explain the magnitude of beta diversity differentiation across sites (Table 6), as the two furthest sites (Mangevo and Vohiparara) were more similar than sites in closer geographic proximity. In fact, for both weighted metrics (weighted UniFrac and Bray–Curtis), Mangevo and Vohiparara were not statistically different (Table 6; Table S4).

**Table 6:** No clear effect of geographic distance on gut microbiome beta diversity.

Site	Vohiparara	Talatakely	Valohoaka	Mangevo
Vohiparara	x	3.78*	6.28*	4.58*
Talatakely	8.04*	x	3.68*	3.13*
Valohoaka	4.86*	2.53	x	4.13*
Mangevo	2.09	15.39*	4.37*	x

*Note:* Results of pairwise PERMANOVA tests of beta diversity for the independent variable sampling site using the mean‐ceiling data set. Sites are arranged from north to south in the header row and column (see Figure 1 and Table 1). To show the magnitude of microbiome difference between sites, cells denote pseudo‐F values.

* Significant *q*‐values (*q* < 0.05). The top half of the matrix (shaded light orange) shows unweighted UniFrac values; the bottom half (shaded light gray) shows weighted UniFrac. For both metrics, the magnitude of differentiation does not increase with distance (i.e., Vohipara and Mangevo are not the most different).

**Figure 4:**
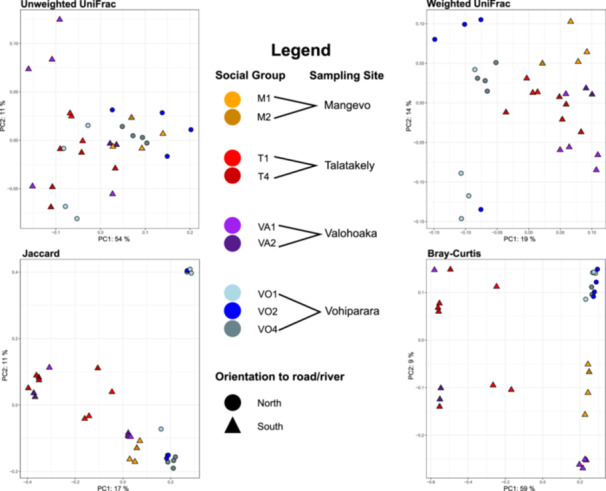
PCoA plots of microbiome variation across four beta diversity metrics using the mean‐ceiling data set. Shape corresponds to orientation to the road/river and color corresponds with social group and site (e.g., all shades of orange denote Mangevo). In all but one plot (unweighted UniFrac), samples strongly segregate based orientation to the road/river. Samples also cluster by social group and site, with varying degrees of overlap across beta diversity metrics.

PCoA plots tended to show separation of samples north and south of the road/river, though this distinction was not well‐defined using Weighted UniFrac. Clustering was also evident with respect to sampling site and group identity for all plots, though samples had some degree of overlap (Figure 4). Samples from Vohiparara (north of the road/river) exhibited the greatest intra‐population dispersion (Figure 4).

## 4 Discussion

This study is the first to systematically examine the gut microbiome of *P. edwardsi*, an endangered flagship species. We found the greatest beta diversity differentiation to occur between social groups, with significant divisions on opposite sides of geographic barriers (road/river). Habitat disturbance had the most significant effect on alpha diversity, though, contrary to expectations, disturbance was associated with higher microbial species richness. Finally, we found that geographic distance alone did not strongly impact gut microbiome diversity. In combination, these results demonstrate how anthropogenic and natural forces shape the *P. edwardsi* microbiome, with particularly interesting implications for social animals and gut microbiome biogeography.

### 4.1 The Role of Geographic Barriers in *P. edwardsi* Gut Microbiome Divergence

Social group stood out as the most powerful factor shaping variation in this *P. edwardsi* gut microbiome data set. This pattern likely arose through a combination of familial relatedness (i.e., vertical microbial transmission), shared environment and diet, and within‐group social cohesion (e.g., transfer of microbiota via grooming). The importance of social group fits within a larger framework, as previous work in a congeneric species (*Propithecus verreauxi*) has shown that different social groups harbored distinct gut microbiomes (Rudolph et al. 2022), and that this distinction could be maintained over multiple years despite major shifts in group composition (Perofsky et al. 2021).

Our study adds a new dimension by comparing gut microbiome variation of populations on either side of a major barrier to animal movement. We found that groups in Vohiparara (north of road/river) were highly distinct from groups in the south. This suggests abrupt dispersal limitation exacerbates gut microbiome divergence, even on small geographic scales. Interestingly, our findings in gut microbiota closely mirror patterns reported in *P. edwardsi* population genetics. Using microsatellite data and leveraging these same populations, Morelli (2008) uncovered significant population structure between groups north and south of the road/river. However, despite these genetic differences, there was still some degree of admixture between *P. edwardsi* in Vohiparara and the south. The presence of admixture excludes the Namorona River as an impenetrable barrier to gene flow, which is reasonable given the river (1) has flowed for millions of years (Wang et al. 2021); (2) is relatively narrow, especially during the dry season; and (3) does not represent a species boundary—an evolutionarily important point, as *Propithecus* species ranges are often bound by rivers (Goodman and Ganzhorn 2003; Pearson and Raxworthy 2009). Thus, a moderate level of *P. edwardsi* migrants have likely traversed both sides of the river over millenia (Morelli 2008).

The effects of the road are more poorly understood. Despite being framed with deforestation and heavily trafficked, some Malagasy species have been observed crossing the road, including *Eulemur rufifrons* and tenrecs (Donohue, pers. obs). The road's effect on *P. edwardsi* movement remains unclear, though many individuals in RNP are collared and continuously followed by a long‐term research team, so dispersal between the north and south would have a high likelihood of being recorded. Furthermore, the highway has a much younger genesis—established in the 1930s and paved in the 1960s—ensuring its effect on the genomes of long‐lived species like *Propithecus* may take generations to realize.

Nonetheless, because the gut microbiome evolves more quickly than the host genome, it allows for more rapid detection of evolutionary trends, including incipient population divergence and adaptation (Alberdi et al. 2016). Thus, the gut microbiome may signal effects of the road long before genomic evidence arises. To understand the relative timing of microbiome divergence in our data set, we compared results between phylogenetic beta diversity metrics, which weight older microbial clades, and taxonomic metrics, which are more sensitive to nascent clades (Sanders et al. 2013; Donohue et al. 2022). We found the highest differentiation between the north and south using a taxonomic metric (Bray–Curtis), which suggests many of the microbes driving microbiome divergence between the north and south were acquired recently in evolutionary time (Table 4). Though we cannot calibrate the timing of actual gut microbiome divergence, it is interesting to consider this pattern in light of the more recent construction of the road relative to the river.

Overall, our findings echo previous reports of Primate microbiome divergence across nascent geographic barriers. In a seminal example, arboreal red colobus monkeys exhibited significant microbiome divergence across an area spanning just 13 km, due in large part to forest fragmentation (Barelli et al. 2015). A greater body of work has compared gut microbiome patterns over large distances. For example, significant effects of geography were also recovered across wide sampling scales for chimpanzees (de Mesquita et al. 2021), gorillas (Gomez et al. 2015), and ring‐tailed lemurs (Bennett et al. 2016). However, Grieneisn et al. (2019) detected no effect of geography across a 375 km transect of hybrid baboons. Notoriously, human gut microbiome variation is also not well explained by geography (e.g., T. P. Campbell et al. 2020). In our data set, geographic distance was not the best explainer of microbiome variation, though it is possible our results would have been different had we sampled a larger area. Thus, effects of geography are likely taxon, scale, and context‐dependent.

### 4.2 The Effects of Habitat Disturbance on the *P. edwardsi* Gut Microbiome

As part of our study design, we sampled *P. edwardsi* across sites with different histories of habitat disturbance. All sites are in the same national park, encompassing a straight‐line distance of less than 20 km. Despite this small geographic scale, and three of the four sites being part of the same continuous forest block, we found that gut microbiomes from sites with histories of habitat disturbance differed significantly compared with those in sites which were never logged. Habitat disturbance had the second‐largest effect size for beta diversity (first being orientation to road/river; Table 4), and it was the only independent variable in our study to significantly impact alpha diversity (Figure 4). We also identified five microbial genera that were more abundant in disturbed than pristine habitats (Table 1). Together, these results offer compelling new evidence about how deforestation—even after 30 years of forest regrowth—can change host‐microbial symbioses in endangered Primates.

Gut microbiome diversity patterns cannot be used alone to assess host health, as one is not able to determine whether differences are adaptive, deleterious, or neutral. In studies that found an effect of disturbance, most reported lower gut microbiome alpha diversity in more disturbed sites (e.g., Amato et al. 2013; Bennett et al. 2016; Trosvik et al. 2018; Donohue et al. 2019; Wasimuddin et al. 2017). Our study found the opposite pattern, with individuals in disturbed sites harboring more species‐rich microbiomes (Figure 4). This presents an interesting parallel to captive *Propithecus*, which were found to exhibit greater alpha diversity than wild counterparts (Greene et al. 2021). Therefore, it is possible that environmental stress may increase gut microbial diversity in sifakas, though more research is needed. Typically, increased alpha diversity is associated with consuming more diverse foods (e.g., Amato et al. 2013; Barelli et al. 2015; McManus et al. 2021). However, diet does not offer the best explanation for our findings. A concurrent behavioral study of *P. edwardsi* at the two most distantly spaced sites—Vohiparara (disturbed) and Mangevo (pristine)—reported similar but slightly lower dietary diversity in Vohiparara (12 food genera, vs. 13 in Mangevo) (Matos, Fernandes, and Wright 2022). This study also found that though taxonomic composition of food items varied between sites, overall dietary guild (i.e., proportion of leaves, fruits, and seeds) did not (Matos, Fernandes, and Wright 2022). Furthermore, samples across sites demonstrated hallmark signatures of fruit‐rich diets, including high abundance of Bacteroidetes and Firmicutes (Figure 2), which are associated with protein and fiber, respectively (Sun et al. 2016; Greene et al. 2021).

Notably, another concurrent study of *P. edwardsi* in three of our four sites (Vohiparara, Talatakely, and Valohoaka) reported significantly less time was devoted to grooming in disturbed sites, with no other behavioral differences (Krauss 2018). With all other aspects of activity budget being statistically identical, it is possible that reduced grooming could be a vestigial adaptation to cope with habitat disturbance, as Vohiparara and Talatakely have been well protected for over 30 years (Krauss 2018). This hypothesis may be supported given the context of past work showing sifakas in disturbed forests tend to spend less time engaged in social behaviors and more time resting (Arrigo‐Nelson 2006).

Reduction in grooming could explain the higher dispersion of microbiome beta diversity in disturbed sites, as affiliative interactions tend to increase microbiome homogenization among groupmates (e.g., Perofsky et al. 2021). It is also possible that reduced grooming of *P. edwardsi* in disturbed habitats is an adaptation to help avoid transmission of antagonistic microbes, as our data provide some evidence of dysbiosis in disturbed populations. First, beta diversity of samples from disturbed sites, particularly Vohiparara, had greater intra‐population variability and dispersion (Figure 3). This is significant because the Anna Karenina Principle posits that dysbiotic hosts are more likely to exhibit high within‐group variation (Zaneveld, McMinds, and Vega Thurber 2017). However, Vohiparara could have such high intra‐population variability simply because it has substantially more samples than the other sites. Second, ANCOM results identified two potential biomarkers of dysbiosis and/or disease in disturbed habitats: *Bacteroides* (Wexler and Goodman 2017; Clayton et al. 2018) and *Anaerostipes* (Zhang et al. 2023; Liu et al. 2021) (Table 1).

Of course, this hypothesis is debatable. Disturbed sites also had increased abundance of *Coprobacillus*, which is not associated with dysbiosis (Shi et al. 2018). Furthermore, *Prevotella*, an indicator of poor gut health (Clayton et al. 2018), was most abundant in Valohoaka, a pristine site. Additionally, although microbial dispersion was most extreme in Vohiparara, it was also evident in the Jaccard plots of Talatakely (another disturbed site) and Valohoaka (pristine) (Figure 3).

Though we can reasonably conclude habitat disturbance impacts gut microbiome diversity and composition, the data at hand only allow us to speculate about its overall meaning for the health, conservation, and evolution of this endangered species. Future work should prioritize pairing gut microbiome data with biomedical, behavioral, and ecological data to better assess the health of *P. edwardsi* in the Ranomafana habitats.

### 4.3 Limitations

Though most of the samples collected were from well‐studied groups, three (VO3 and both Mangevo groups) were unhabituated and previously unknown to the Centre ValBio research team. The high degree of difficulty sampling these groups yielded fewer samples, leading to uneven sample sizes across sites and a particularly low sample size in Mangevo. This reduction in statistical power could meaningfully impact our results, as it may lower the probability of finding true biological effects and/or impact the effect size of significant relationships (Button et al. 2013; Makin and Orban de Xivry 2019).

### 4.4 Conclusions and Future Directions

This study showed that the road/river and habitat disturbance significantly impacted the *P. edwardsi* gut microbiome in RNP. Based on these initial results, we highlight the need for additional research regarding health outcomes of gut microbiome divergence in disturbed/undisturbed and northern/southern sites. We also recommend greater sampling effort at Mangevo and across the *P. edwardsi* range more broadly, including sites in the COFAV corridor and Andringitra National Park, where they were recently sighted after being absent for over 20 years (Batist et al. 2023).

We present two hypotheses which can be tested with repeated sampling of RNP's *P. edwardsi* over time. First, we hypothesize that gut microbiome divergence will continue to intensify between RNP's northern and southern *Propithecus* populations without conservation intervention, such as the construction of wildlife bridges. The data presented herein serve as a baseline that should be used for evaluating the efficacy of conservation interventions, not only for gut microbiomes but also migration and gene flow, as migratory events can be difficult to detect behaviorally, and genomic impacts take many generations to materialize. With increased migration and gene flow, gut microbiomes may homogenize. Second, we hypothesize that differences associated with habitat disturbance will decrease over time as the forest recovers. To further test this hypothesis, we recommend pairing microbiome sampling with detailed ecological assessments and botanical surveys of each site at multiple time points over the coming decades.

The gut microbiome has great, largely unrealized potential as a tool for monitoring endangered species such as *P. edwardsi*, and we hope this study has laid a foundation for more comprehensive, long‐term research. We also encourage additional work, both within Primates and across the Tree of Life, examining how anthropogenic barriers such as roads and cities impact gut microbiome divergence—and its consequences—between isolated populations.

## Author Contributions

**Mariah E. Donohue:** conceptualization (equal), formal analysis (lead), methodology (equal), validation (lead), visualization (lead), writing–original draft (equal), writing–review and editing (equal). **Alicia Lamb:** conceptualization (equal), data curation (equal), funding acquisition (equal), investigation (lead), project administration (lead), writing–original draft (equal), writing–review & editing (equal). **Abigail E. Absangba:** data curation (equal), methodology (equal), validation (equal), writing–review and editing (equal). **Eliette Noromalala:** investigation (supporting), methodology (supporting), project administration (supporting), writing–review and editing (equal). **David R. Weisenbeck:** formal analysis (supporting), visualization (equal), writing–review and editing (equal). **Rebecca M. Stumpf:** funding acquisition (equal), resources (lead), writing–review and editing (equal). **Patricia C. Wright:** conceptualization (equal), funding acquisition (supporting), resources (equal), supervision (lead), writing–review and editing (equal).

## Conflicts of Interest

Patricia C. Wright is on the advisory board of RW Primate Fund, one of the funders of this project. She did not advise on funding this project.

## Supporting information

Supporting information.

## Data Availability

Sequence data are available under BioProject ID PRJNA119305 at http://www.ncbi.nlm.nih.gov/bioproject/1193052.

## References

[ajp23732-bib-0001] Alberdi, A. , O. Aizpurua , K. Bohmann , M. L. Zepeda‐Mendoza , and M. T. P. Gilbert . 2016. “Do Vertebrate Gut Metagenomes Confer Rapid Ecological Adaptation?” Trends in Ecology & Evolution 31, no. 9: 689–699. 10.1016/j.tree.2016.06.008. 27453351

[ajp23732-bib-0002] Amato, K. R. , J. G. Sanders , S. J. Song , et al. 2019. “Evolutionary Trends in Host Physiology Outweigh Dietary Niche in Structuring Primate Gut Microbiomes.” ISME Journal 13: 576–587. 10.1038/s41396-018-0175-0. 29995839 PMC6461848

[ajp23732-bib-0003] Amato, K. R. , C. J. Yeoman , A. Kent , et al. 2013. “Habitat Degradation Impacts Black Howler Monkey (*Alouatta pigra*) Gastrointestinal Microbiomes.” ISME Journal 7: 1344–1353. 10.1038/ismej.2013.16. 23486247 PMC3695285

[ajp23732-bib-0004] Arrigo‐Nelson, S. J. (2006). The Impact of Habitat Disturbance on the Feeding Ecology of the Milne‐Edwards’ Sifaka (*Propithecus edwardsi*) in Ranomafana National Park, Madagascar. PhD thesis, State University of New York, Stony Brook.

[ajp23732-bib-0005] Barelli, C. , D. Albanese C. Donati , et al. 2015. “Habitat Fragmentation Is Associated to Gut Microbiota Diversity of an Endangered Primate: Implications for Conservation.” Scientific Reports 5: 14862. 10.1038/srep14862. 26445280 PMC4595646

[ajp23732-bib-0006] Batist, C. H. , M. Bliss , D. R. Weisenbeck , et al. 2023. “Updated Lemur Species Ranges in Madagascar's Corridor Forestier d'ambositra Vondrozo (COFAV).” Folia Primatologica 1: 1–17. 10.1163/14219980-bja10012. 38593416

[ajp23732-bib-0007] Bennett, G. , M. Malone , M. L. Sauther , et al. 2016. “Host Age, Social Group, and Habitat Type Influence the Gut Microbiota of Wild Ring‐Tailed Lemurs (*Lemur catta*).” American Journal of Primatology 78, no. 8: 883–892. 10.1002/ajp.22555. 27177345

[ajp23732-bib-0009] Bolyen, E. , J. R. Rideout , M. R. Dillon , et al. 2019. “Reproducible, Interactive, Scalable and Extensible Microbiome Data Science Using QIIME 2: Reproducible, Interactive, Scalable, and Extensive Microbiome Data Science.” Nature Biotechnology 37: 852–857. 10.1038/s41587-019-0209-9. 31399723

[ajp23732-bib-0010] Brooks, A. W. , K. D. Kohl , R. M. Brucker , E. J. van Opstal , and S. R. Bordenstein . 2016. “Phylosymbiosis: Relationships and Functional Effects of Microbial Communities Across Host Evolutionary History.” PLoS Biology 14, no. 11: e2000225. 10.1371/journal.pbio.2000225. 27861590 PMC5115861

[ajp23732-bib-0011] Brunke, J. , U. Radespiel , I. R. Russo , M. W. Bruford , and B. Goossens . 2019. “Messing About on the River: The Role of Geographic Barriers in Shaping the Genetic Structure of Bornean Small Mammals in a Fragmented Landscape.” Conservation Genetics 20: 691–704. 10.1007/s10592-019-01159-3.

[ajp23732-bib-0012] Button, K. S. , J. P. A. Ioannidis , C. Mokrysz , et al. 2013. “Power Failure: Why Small Sample Size Undermines the Reliability of Neuroscience.” Nature Reviews Neuroscience 14: 365–376. 10.1038/nrn3475. 23571845

[ajp23732-bib-0013] Callahan, B. J. , P. J. McMurdie , M. J. Rosen , A. W. Han , A. J. A. Johnson , and S. P. Holmes . 2016. “DADA2: High‐Resolution Sample Inference Form Illumina Amplicon Data.” Nature Methods 13: 581–583. 10.1038/nmeth.3869. 27214047 PMC4927377

[ajp23732-bib-0014] Campbell, J. L. , J. H. Eisemann , C. V. Williams , and K. M. Glenn . 2000. “Description of the Gastrointestinal Tract of Five Lemur Species: *Propithecus tattersalli*, *Propithecus verreauxi coquereli*, *Varecia variegata*, *Hapalemur griseus*, and *Lemur catta* .” American Journal of Primatology 52: 133–142. 10.1002/1098-2345(200011)52:3<133::AID-AJP2>3.0.CO;2-#. 11078027

[ajp23732-bib-0070] Hansen, M. C. , P. V. Potapov , R. Moore , et al. 2013. “High‐Resolution Global Maps of 21st‐Century Forest Cover Change.” Science 342, no. 6160: 850–853. 10.1126/science.1244693. 24233722

[ajp23732-bib-0015] Campbell, T. P. , X. Sun , V. H. Patel , C. Sanz , D. Morgan , and G. Dantas . 2020. “The Microbiome and Resistome of Chimpanzees, Gorillas, and Humans Across Host Lifestyle and Geography.” ISME Journal 14: 1584–1599. 10.1038/s41396-020-0634-2. 32203121 PMC7242348

[ajp23732-bib-0016] Clayton, J. B. , A. Gomez , K. Amato , et al. 2018. “The Gut Microbiome of Nonhuman Primates: Lessons in Ecology and Evolution.” American Journal of Primatology 80, no. 6: e22867. 10.1002/ajp.22867. 29862519

[ajp23732-bib-0017] Clough, D. , M. Heistermann , and P. M. Kappeler . 2010. “Host Intrinsic Determinants and Potential Consequences of Parasite Infection in Free‐Ranging Red‐Fronted Lemurs (*Eulemur fulvus rufus*).” American Journal of Biological Anthropology 142, no. 3: 441–452. 10.1002/ajpa.21243. 20091843

[ajp23732-bib-0018] Donohue, M. E. , A. E. Asangba , J. Ralainirina , D. W. Weisrock , R. M. Stumpf , and P. C. Wright . 2019. “Extensive Variability in the Gut Microbiome of a Highly‐Specialized and Critically Endangered Lemur Species Across Sites.” American Journal of Primatology 81, no. 10–11: e23046. 10.1002/ajp.23046. 31478232

[ajp23732-bib-0019] Donohue, M. E. , Z. L. Hert , C. E. Karrick , et al. 2023. “Lemur Gut Microeukaryotic Community Variation Is Not Associated With Host Phylogeny, Diet, or Habitat.” Microbial Ecology 86: 2149–2160. 10.1007/s00248-023-02233-7. 37133496

[ajp23732-bib-0020] Donohue, M. E. , A. K. Rowe , E. Kowalewski , et al. 2022. “Significant Effects of Host Dietary Guild and Phylogeny in Wild Lemur Gut Microbiomes.” ISME Communications 2: 33. 10.1038/s43705-022-00115-6. 37938265 PMC9723590

[ajp23732-bib-0021] Gerber, B. D. , S. Arrigo‐Nelson , S. M. Karpanty , M. Kotschwar , and P. C. Wright . 2012. “Spatial Ecology of the Endangered Milne‐Edwards’ Sifaka (*Propithecus edwardsi*): Do Logging and Season Affect Home Range and Daily Ranging Patterns?” International Journal of Primatology 33: 305–321. 10.1007/s10764-011-9576-xs.

[ajp23732-bib-0022] Gomez, A. , K. Petrzelkova , C. J. Yeoman , et al. 2015. “Gut Microbiome Composition and Metabolomic Profiles of Wild Western Lowland Gorillas (*Gorilla gorilla gorilla*) Reflect Host Ecology.” Molecular Ecology 24, no. 10: 2551–2565. 10.1111/mec.13181. 25846719

[ajp23732-bib-0023] Goodman, S. M. , and J. U. Ganzhorn . 2003. “Biogeography of Lemurs in the Humid Forests of Madagascar: The Role of Elevational Distribution and Rivers.” Journal of Biogeography 31, no. 1: 47–55. 10.1111/j.1365-2699.2004.00953.x.

[ajp23732-bib-0024] Greene, L. K. , M. B. Blanco , and E. Rambeloson , et al. 2021. “Gut Microbiota of Frugo‐Folivorous Sifakas Across Environments.” Animal Microbiome 3, no. 39: 39. 10.1186/s42523-021-00093-5. 34006323 PMC8132362

[ajp23732-bib-0025] Greene, L. K. , S. L. Bornbusch , E. A. McKenney , et al. 2019. “The Importance of Scale in Comparative Microbiome Research: New Insights From the Gut and Glands of Captive and Wild Lemurs.” American Journal of Primatology 81, no. 10–11: e22974. 10.1002/ajp.22974. 30932230

[ajp23732-bib-0026] Greene, L. K. , J. B. Clayton , R. S. Rothman , et al. 2019. “Local Habitat, Not Phylogenetic Relatedness, Predicts Gut Microbiota Better Within Folivorous Than Frugivorous Lemur Lineages.” Biology Letters 15, no. 6: 20190028. 10.1098/rsbl.2019.0028. 31185820 PMC6597504

[ajp23732-bib-0027] Greene, L. K. , E. A. McKenney , W. Gasper , et al. 2023. “Gut Site and Gut Morphology Predict Microbiome Structure and Function in Ecologically Diverse Lemurs.” Microbial Ecology 85: 1608–1619. 10.1007/s00248-022-02034-4. 35562600

[ajp23732-bib-0028] Greene, L. K. , C. V. Williams , R. E. Junge , et al. 2020. “A Role for Gut Microbiota in Host Niche Differentiation.” ISME Journal 14: 1675–1687. 10.1038/s41396-020-0640-4. 32238913 PMC7305313

[ajp23732-bib-0029] Grieneisn, L. E. , M. J. E. Charpentier , S. C. Alberts , et al. 2019. “Genes, Geology and Germs: Gut Microbiota Across a Primate Hybrid Zone Are Explained by Site Soil Properties, Not Host Species.” Proceedings of the Royal Society B 286, no. 1901: 20190431. 10.1098/rspb.2019.0431. 31014219 PMC6501927

[ajp23732-bib-0031] Krauss, J. 2018. “Effects of Past Logging on Diet and Behaviour of Milne Edward's Sifaka in Ranomafana National Park.” Lemur News 21: 36–38.

[ajp23732-bib-0032] Liu, X. , Y. W. Cheng , L. Shao , et al. 2021. “Gut Microbiota Dysbiosis in Chinese Children With Type 1 Diabetes Mellitus: An Observational Study.” World Journal of Gastroenterology 27, no. 19: 2394–2414. 10.3748/wjg.v27.i19.2394. 34040330 PMC8130045

[ajp23732-bib-0033] Maigret, T. A. , J. J. Cox , and D. W. Weisrock . 2020. “A Spatial Genomic Approach Identifies Time Lags and Historical Barriers to Gene Flow in a Rapidly Fragmenting Appalachian Landscape.” Molecular Ecology 29, no. 4: 673–685. 10.1111/mec.15362. 31981245

[ajp23732-bib-0034] Makin, T. R. , and J. J. Orban de Xivry . 2019. “Science Forum: Ten Common Statistical Mistakes to Watch Out for When Writing or Reviewing a Manuscript.” eLife 8: e48175. 10.7554/eLife.48175. 31596231 PMC6785265

[ajp23732-bib-0035] Mandal, S. , W. van Treuren , R. A. White , M. Eggesbø , R. Knight , and S. D. Peddada . 2015. “Analysis of Composition of Microbiomes: A Novel Method for Studying Microbial Composition.” Microbial Ecology in Health and Disease 26, no. 1: 27663. 10.3402/mehd.v26.27663. 26028277 PMC4450248

[ajp23732-bib-0036] Matos, M. D. P. , T. R. M. Fernandes , and P. C. Wright . 2022. “Ecological Flexibility of *Propithecus edwardsi* in Two Forest Habitats With Different Logging Histories in Ranomafana National Park, Madagascar.” International Journal of Primatology 43: 913–931. 10.1007/s10764-022-00308-9.

[ajp23732-bib-0037] McKenney, E. A. , A. Rodrigo , and A. D. Yoder . 2015. “Patterns of Gut Bacterial Colonization in Three Primate Species.” PLoS One 10, no. 5: e0124618. 10.1371/journal.pone.0124618. 25970595 PMC4430486

[ajp23732-bib-0038] McManus, N. , S. M. Holmes , E. E. Louis , S. E. Johnson , A. L. Baden , and K. R. Amato . 2021. “The Gut Microbiome as an Indicator of Habitat Disturbance in a Critically Endangered Lemur.” BMC Ecology and Evolution 21: 222. 10.1186/s12862-021-01945-z. 34915861 PMC8680155

[ajp23732-bib-0039] de Mesquita, C. P. , L. M. Nichols , M. J. Gebert , et al. 2021. “Structure of Chimpanzee Gut Microbiomes Across Tropical Africa.” mSystems 6: e01269‐20. 10.1128/mSystems.01269-20. PMC8269259 34156289

[ajp23732-bib-0040] Morelli, T. L. (2008). *Dispersal, Kinship, and Genetic Structure of an Endangered Madagascar Primate, Propithecus edwardsi*. PhD thesis, State University of New York, Stony Brook.

[ajp23732-bib-0041] Morelli, T. L. , S. J. King , S. T. Pochron , and P. C. Wright . 2009. “The Rules of Disengagement: Takeovers, Infanticide, and Dispersal in a Rainforest Lemur, *Propithecus edwardsi* .” Behaviour 146, no. 4/5: 499–523. 10.1163/15683908X399554.

[ajp23732-bib-0042] Murillo, T. , D. Schneider , C. Fichtel , and R. Daniel . 2022. “Dietary Shifts and Social Interactions Drive Temporal Fluctuations of the Gut Microbiome From Wild Redfronted Lemurs.” ISME Communications 2: 3. 10.1038/s43705-021-00086-0. 37938637 PMC9723586

[ajp23732-bib-0043] Pearson, R. G. , and C. J. Raxworthy . 2009. “The Evolution of Local Endemism in Madagascar: Watershed Versus Climatic Gradient Hypotheses Evaluated by Null Biogeographic Models.” Evolution 63, no. 4: 959–967. 10.1111/j.1558-5646.2008.00596.x. 19210532

[ajp23732-bib-0044] Peixoto, R. S. , D. M. Harkins , and K. E. Nelson . 2021. “Advances in Microbiome Research for Animal Health.” Annual Review of Animal Biosciences 9: 289–311. 10.1146/annurev-animal-091020-075907. 33317323

[ajp23732-bib-0045] Perofsky, A. C. , L. Ancel Meyers , L. A. Abondano , A. Di Fiore , and R. J. Lewis . 2021. “Social Groups Constrain the Spatiotemporal Dynamics of Wild Sifaka Gut Microbiomes.” Molecular Ecology 30, no. 24: 6759–6775. 10.1111/mec.16193. 34558751 PMC8665069

[ajp23732-bib-0046] Perofsky, A. C. , R. J. Lewis , and L. A. Meyers . 2019. “Terrestriality and Bacterial Transfer: A Comparative Study of Gut Microbiomes in Sympatric Malagasy Mammals.” ISME Journal 13: 50–63. 10.1038/s41396-018-0251-5. 30108305 PMC6299109

[ajp23732-bib-0047] Pochron, S. T. , J. Fitzgerald , C. C. Gilbert , et al. 2003. “Patterns of Female Dominance in *Propithecus diadema edwardsi* of Ranomafana National Park, Madagascar.” American Journal of Primatology 61, no. 4: 173–185. 10.1002/ajp.10119. 14669269

[ajp23732-bib-0071] Pochron, S. T. , W. T. Tucker , and P. C. Wright . 2004. “Demography, Life History, and Social Structure in Propithecus Edwardsi From 1986‐2000 in Ranomafana National Park, Madagascar.” American Journal of Biological Anthropology 125, no. 1: 61–72. 10.1002/ajpa.10266. 15293332

[ajp23732-bib-0048] Quast, C. , E. Pruesse , and P. Yilmaz , et al. 2012. “The SILVA Ribosomal RNA Gene Database Project: Improved Data Processing and Web‐Based Tools.” Nucleic Acids Research 41, no. D1: D590–D596. 10.1093/nar/gks1219. 23193283 PMC3531112

[ajp23732-bib-0049] Raulo, A. , L. Ruokolainen , A. Lane , et al. 2017. “Social Behaviour and Gut Microbiota in Red‐Bellied Lemurs (*Eulemur rubriventer*): In Search of the Role of Immunity in the Evolution of Sociality.” Journal of Animal Ecology 87, no. 2: 388–399. 10.1111/1365-2656.12781. 29205327

[ajp23732-bib-0050] Rowe, A. K. , M. E. Donohue , E. L. Clare , et al. 2021. “Exploratory Analysis Reveals Arthropod Consumption in 10 Lemur Species Using DNA Metabarcoding.” American Journal of Primatology 83, no. 6: e23256. 10.1002/ajp.23256. 33818786

[ajp23732-bib-0051] Rudolph, K. , D. Schneider , C. Fichtel , R. Daniel , M. Heistermann , and P. M. Kappeler . 2022. “Drivers of Gut Microbiome Variation Within and Between Groups of a Wild Malagasy Primate.” Microbiome 10, no. 28: 28. 10.1186/s40168-021-01223-6. 35139921 PMC8827170

[ajp23732-bib-0052] Rundle, H. D. , and P. Nosil . 2005. “Ecological Speciation.” Ecology Letters 8, no. 3: 336–352. 10.1111/j.1461-0248.2004.00715.x.

[ajp23732-bib-0072] Sanders, J. G. , S. Powell , D. J. C. Kronauer , et al. 2013. “Stability and Phylogenetic Correlation in Gut Microbiota: Lessons From Ants and Apes.” Molecular Ecology 23, no. 6: 1268–1283. 10.1111/mec.12611 24304129

[ajp23732-bib-0053] Shi, Y. , L. Kellingray , Q. Zhai , et al. 2018. “Structural and Functional Alterations in the Microbial Community and Immunological Consequences in a Mouse Model of Antibiotic‐Induced Dysbiosis.” Frontiers in Microbiology 9. 10.3389/fmicb.2018.01948. PMC6110884 30186263

[ajp23732-bib-0054] Stumpf, R. M. , A. Gomez , K. R. Amato , et al. 2016. “Microbiomes, Metagenomics, and Primate Conservation: New Strategies, Tools, and Applications.” Biological Conservation 199: 56–66. 10.1016/j.biocon.2016.03.035.

[ajp23732-bib-0055] Sun, B. , X. Wang , S. Bernstein , et al. 2016. “Marked Variation Between Winter and Spring Gut Microbiota in Free‐Ranging Tibetan Macaques (*Macaca tibetan)* .” Scientific Reports 6: 26035. 10.1038/srep26035. 27180722 PMC4867428

[ajp23732-bib-0056] Trosvik, P. , E. K. Rueness , E. J. de Muinck , A. Moges , and A. Mekonnen . 2018. “Ecological Plasticity in the Gastrointestinal Microbiomes of Ethiopian *Chlorocebus* Monkeys.” Scientific Reports 8, no. 20: 20. 10.1038/s41598-017-18435-2. 29311667 PMC5758796

[ajp23732-bib-0057] Umanets, A. , I. de Winter , F. IJdema , et al. 2018. “Occupancy Strongly Influences Faecal Microbial Composition of Wild Lemurs.” FEMS Microbiology Ecology 94, no. 3: fiy017. 10.1093/femsec/fiy017. 29415151

[ajp23732-bib-0058] Wang, Y. , S. D. Willett , D. Wu , N. Haghipour , and M. Christl . 2021. “Retreat of the Great Escarpment of Madagascar From Geomorphic Analysis and Cosmogenic ^10^Be Concentrations.” Geochemistry, Geophysics, Geosystems 22, no. 12: e2021GC009979. 10.1029/2021GC009979.

[ajp23732-bib-0059] Wasimuddin, G. , S. Menke , J. Melzheimer , et al. 2017. “Gut Microbiomes of Free‐Ranging and Captive Namibian Cheetahs: Diversity, Putative Functions and Occurrence of Potential Pathogens.” Molecular Ecology 26, no. 20: 5515–5527. 10.1111/mec.14278. 28782134

[ajp23732-bib-0060] West, A. G. , D. W. Waite , P. Deines , et al. 2019. “The Microbiome in Threatened Species Conservation.” Biological Conservation 229: 85–98. 10.1016/j.biocon.2018.11.016.

[ajp23732-bib-0061] Wexler, A. G. , and A. L. Goodman . 2017. “An Insider's Perspective: *Bacteroides* as a Window Into the Microbiome.” Nature Microbiology 2: 17026. 10.1038/nmicrobiol.2017.26. PMC5679392 28440278

[ajp23732-bib-0062] Wickham, H. 2009. ggplot2: Elegant Graphics for Data Analysis. Springer New York.

[ajp23732-bib-0063] de Winter, I. I. , A. Umanets , G. Gort , et al. 2020. “Effects of Seasonality and Previous Logging on Faecal Helminth‐Microbiota Associations in Wild Lemurs.” Scientific Reports 10: 16818. 10.1038/s41598-020-73827-1. 33033341 PMC7544911

[ajp23732-bib-0064] Worsley, S. F. , C. S. Davies , M. E. Mannarelli , et al. 2021. “Gut Microbiome Composition, Not Alpha Diversity, Is Associated With Survival in a Natural Vertebrate Population.” Animal Microbiome 3, no. 84: 84. 10.1186/s42523-021-00149-6. 34930493 PMC8685825

[ajp23732-bib-0065] Wright, P. C. 1995. “Demography and Life History of Free‐Ranging *Propithecus diadema edwardsi* in Ranomafana National Park, Madagascar.” International Journal of Primatology 16: 835–854. 10.1007/BF02735722.

[ajp23732-bib-0066] Wright, P. C. 1998. “Impact of Predation Risk on the Behaviour of *Propithecus diadema edwardsi* in the Rainforest of Madagascar.” Behaviour 135, no. 4: 483–512.

[ajp23732-bib-0067] Wright, P. C. , E. M. Erhart , S. Tecot , et al. 2011. “Long‐Term Lemur Research at Centre ValBio, Ranomafana National Park, Madagascar.” In Long‐Term Field Studies of Primates, edited by P. Kappeler and D. Watts . Berlin, Heidelberg, Germany: Springer. 10.1007/978-3-642-22514-7_4.

[ajp23732-bib-0068] Zaneveld, J. R. , R. McMinds , and R. Vega Thurber . 2017. “Stress and Stability: Applying the Anna Karenina Principle to Animal Microbiomes.” Nature Microbiology 2: 17121. 10.1038/nmicrobiol.2017.121. 28836573

[ajp23732-bib-0069] Zhang, W. , X. Xu , L. Cai , and X. Cai . 2023. “Dysbiosis of the Gut Microbiome in Elderly Patients With Hepatocellular Carcinoma.” Scientific Reports 13: 7797. 10.1038/s41598-023-34765-w. 37179446 PMC10182990

