# Supplementary material for: Why Didn't the Sifaka Cross the Road? Divergence of Propithecus edwardsi Gut Microbiomes Across Geographic Barriers in Ranomafana National Park, Madagascar
Source: Am J Primatol. 2025 Feb 4;87(2):e23732. doi: 10.1002/ajp.23732 (PMC11794673; doi:10.1002/ajp.23732)
Supplement: Supplementary file 1 — Supporting information. [file AJP-87-e23732-s001.pdf]

Table S1: QIIME2 mapping file, including sample metadata.

SampleID	Individual	Age	Sex	Site	Group	Orientation	Disturbance
1_B3_JF_T_4	JF-T-4	Juvenile	F	Talatakely	T4	South	High
2_C5_AF_S_1	AF-S-1	Adult	F	Vohiparara	VO1	North	High
2_B3_JM_V_2	JM-V-2	Juvenile	M	Valohoaka	VA2	South	Low
1_D1_GCGT_T_4	GCGT-T-4	Adult	F	Talatakely	T4	South	High
4_D3	AF-S-2	Adult	F	Vohiparara	VO2	North	High
1_H5_SAF_T_1	SAF-T-1	Subadult	F	Talatakely	T1	South	High
1_D5_SAF_T_1	SAF-T-1	Subadult	F	Talatakely	T1	South	High
2_F6_AF_S_1	AF-S-1	Adult	F	Vohiparara	VO1	North	High
2_A5_AM_V_2	AM-V-2	Adult	M	Valohoaka	VA1	South	Low
2_G6_BGMS_V_1	BGMS-V-1	Adult	F	Valohoaka	VA1	South	Low
1_E5_PO_T_4	PO-T-4	Adult	M	Talatakely	T4	South	High
4_H3	AFM-S-2	Adult	F	Vohiparara	VO2	North	High
3_H3	YO-S-4	Adult	F	Vohiparara	VO4	North	High
2_D1_JF_S_1	JF-S-1	Juvenile	F	Vohiparara	VO1	North	High
4_B3	MT-M-1	Adult	M	Mangevo	M1	South	Low
2_D3_SAF_V_2	SAF-V-2	Subadult	F	Valohoaka	VA2	South	Low
2_H1_YO_S_4	YO-S-4	Adult	F	Vohiparara	VO4	North	Low
2_A3_RS_V_2	RS-V-2	Adult	F	Valohoaka	T4	South	Low
1_H6_SAM_S_1	SAM-S-1	Subadult	M	Vohiparara	VO1	North	High
3_C2_RJ_V_1	RJ-V-1	Adult	M	Valohoaka	VA1	South	Low
2_F1_JM_V_2	JM-V-2	Juvenile	M	Valohoaka	VA2	South	Low
2_B5_RS_V_2	RS-V-2	Adult	F	Valohoaka	VA2	South	Low
4_E1	Y-S-2	Yearling		Vohiparara	VO2	North	High
2_A1_SAM_T_1	SAM-T-1	Subadult	F	Talatakely	T4	South	High
1_H5_SAM_S_1	SAM-S-1	Subadult	M	Vohiparara	VO1	North	High
1_F3_OB_T_1	OB-T-1	Adult	M	Talatakely	T1	South	High
1_E6_SAM_V_1	SAM-V-1	Subadult	M	Valohoaka	VA1	South	Low
1_F1_SAM_V_1	SAM-V-1	Subadult	M	Valohoaka	VA1	South	Low
2_D5_JM_V_2	JM-V-2	Juvenile	M	Valohoaka	VA2	South	Low
2_G5_RJ_V_1	RJ-V-1	Adult	M	Valohoaka	VA1	South	Low
4_C1	YO-S-4	Adult	F	Vohiparara	VO4	North	High
3_B6_RJ_V_1	RJ-V-1	Adult	M	Valohoaka	VA1	South	Low
2_B1_SAF_V_2	SAF-V-2	Subadult	F	Valohoaka	VA2	South	Low
4_B1	AF-M-1	Adult	F	Mangevo	M1	South	Low
4_C3	SR-S-4	Adult	M	Vohiparara	VO4	North	High
4_H1	AF-S-2	Adult	F	Vohiparara	VO2	North	High
1_E5_BGMS_V_1	BGMS-V-1	Adult	F	Valohoaka	VA1	South	Low
1_F1_JF_T_4	JF-T-4	Juvenile	F	Talatakely	T4	South	High
2_G4_PBPS_V_1	PBPS-V-1	Adult	F	Valohoaka	VA1	South	Low
4_G5	Y-S-2	Yearling		Vohiparara	VO2	North	High
1_D3_PO_T_4	PO-T-4	Adult	M	Talatakely	T4	South	High
2_A3_AF_S_4	AF-S-4	Adult	F	Vohiparara	VO4	North	High
1_H3_JF_S_1	JF-S-1	Juvenile	F	Vohiparara	VO1	North	High
4_G1	AM-S-2	Adult	M	Vohiparara	VO2	North	High
1_A5_GCGT_T_4	GCGT-T-4	Adult	F	Talatakely	T4	South	High
1_C1_GCGT_T_4	GCGT-T-4	Adult	F	Talatakely	T4	South	High
2_G3_AF_S_1	AF-S-1	Adult	F	Vohiparara	VO1	North	High
3_A6_OB_T_1	OB-T-1	Adult	M	Talatakely	T1	South	High
1_A3_TR_T_4	TR-T-4	Adult	F	Talatakely	T4	South	High
1_E1_TR_T_4	TR-T-4	Adult	F	Talatakely	T4	South	High

4_G3	AM-S-2	Adult	M	Vohiparara	VO2	North	High
4_F5	AFM-S-2	Adult	F	Vohiparara	VO2	North	High
1_D6_OB_T_1	OB-T-1	Adult	M	Talatakely	T1	South	High
4_A1	MT-M-1	Adult	M	Mangevo	M1	South	Low
2_A1_SAM_V_1	SAM-V-1	Subadult	M	Valohoaka	VA1	South	Low
2_A2_PBPS_V_1	PBPS-V-1	Adult	F	Valohoaka	VA1	South	Low
1_A1_Y_S_2	Y-S-2	Yearling	NA	Vohiparara	VO4	North	High
3_C3_SAM_S_4	SAM-S-4	Subadult	M	Vohiparara	VO4	North	High
2_D1_AM_V_2	AM-V-2	Adult	M	Valohoaka	VA2	South	Low
1_B5_TR_T_4	TR-T-4	Adult	F	Talatakely	T4	South	High
1_F5_SAF_T_1	SAF-T-1	Subadult	F	Talatakely	T1	South	High
3_B3_SAF_S_1	SAF-S-1	Subadult	F	Vohiparara	VO1	North	High
3_B1_SAM_T_1	SAM-T-1	Subadult	M	Talatakely	T1	South	High
4_A4	AF-M-1	Adult	F	Mangevo	M1	South	Low
1_H3_OB_T_1	OB-T-1	Adult	M	Talatakely	T1	South	High
1_H2_SAF_T_1	SAF-T-1	Subadult	F	Talatakely	T1	South	High
2_F5_SAF_T_1	SAF-T-1	Subadult	F	Talatakely	T1	South	High
3_B4_AM_S_1	AM-S-1	Adult	M	Vohiparara	VO1	North	High
1_D5_JF_T_4	JF-T-4	Juvenile	F	Talatakely	T4	South	High
3_B5_AM_S_1	AM-S-1	Adult	M	Vohiparara	VO1	North	High
3_C1_BGMS_V_1	BGMS-V-1	Adult	F	Valohoaka	VA1	South	Low
2_G1_AM_S_1	AM-S-1	Adult	M	Vohiparara	VO1	North	High
1_C3_JF_T_4	JF-T-4	Juvenile	F	Talatakely	T4	South	High
3_F5	SR-S-4	Adult	M	Vohiparara	VO4	North	High
1_E1_SAF_S_1	SAF-S-1	Subadult	F	Vohiparara	VO1	North	High
2_C1_AM_V_2	AM-V-2	Adult	M	Valohoaka	VA2	South	Low
3_F1	SAM-S-4	Subadult	M	Vohiparara	VO4	North	High
4_A5	SAF-M-1	Subadult	F	Mangevo	M1	South	Low
2_D2_JF_S_1	JF-S-1	Juvenile	F	Vohiparara	VO1	North	High
1_G3_OB_T_1	OB-T-1	Adult	M	Talatakely	T1	South	High
1_G5_SAF_T_1	SAF-T-1	Subadult	F	Talatakely	T1	South	High
2_E1_RS_V_2	RS-V-2	Adult	F	Valohoaka	VA2	South	Low
3_H5	SAM-S-4	Subadult	M	Vohiparara	VO4	North	High
1_E3_GCGT_T_4	GCGT-T-4	Adult	F	Talatakely	T4	South	High
4_E3	AF-S-2	Adult	F	Vohiparara	VO2	North	High
3_G1	YO-S-4	Adult	F	Vohiparara	VO4	North	High
3_E3	SR-S-4	Adult	M	Vohiparara	VO1	North	High
4_B5	MLG-M-2	Adult	M	Mangevo	M2	South	Low
2_E3_AM_V_2	AM-V-2	Adult	M	Valohoaka	VA2	South	Low
4_F3	AF-S-2	Adult	F	Vohiparara	VO2	North	High
2_C4_SAM_T_1	SAM-T-1	Subadult	M	Talatakely	T1	South	High
3_E1	AF-S-4	Adult	F	Vohiparara	VO1	North	High
1_C5_TR_T_4	TR-T-4	Adult	F	Talatakely	T4	South	High
2_E5_SAF_V_2	SAF-V-2	Subadult	F	Valohoaka	VA2	South	Low
2_D4_BGMS_V_1	BGMS-V-1	Adult	F	Valohoaka	VA1	South	Low
2_D3_PBPS_V_1	PBPS-V-1	Adult	F	Valohoaka	VA1	South	Low
4_E5	AFM-S-2	Adult	F	Vohiparara	VO2	North	High
4_D1	AFM-S-2	Adult	F	Vohiparara	VO2	North	High
2_G2_AF_S_1	AF-S-1	Adult	F	Vohiparara	VO1	North	High
3_E5	YO-S-4	Adult	F	Vohiparara	VO4	North	High
1_E2_SAM_S_1	SAM-S-1	Subadult	M	Vohiparara	VO2	North	High
3_H1	SR-S-4	Adult	M	Vohiparara	VO4	North	High
3_F3	AF-S-4	Adult	F	Vohiparara	VO4	North	High

1_B1_PO_T_4	PO-T-4	Adult	M	Talatakely	T4	South	High
2_C5_RS_V_2	RS-V-2	Adult	F	Valohoaka	VA2	South	Low
1_E4_SAF_S_1	SAF-S-1	Subadult	F	Vohiparara	VO4	North	High
3_B2_AM_S_1	AM-S-1	Adult	M	Vohiparara	VO1	North	High
1_G1_SAM_T_1	SAM-T-1	Subadult	M	Talatakely	T1	South	High
4_D5	AM-S-2	Adult	M	Vohiparara	VO2	North	High
2_C3_OB_T_1	OB-T-1	Adult	M	Talatakely	T1	South	High
3_G5	AF-S-4	Adult	F	Vohiparara	VO4	North	High
1_H4_JF_S_1	JF-S-1	Juvenile	F	Vohiparara	VO1	North	High
3_G3	SAM-S-4	Subadult	M	Vohiparara	VO4	North	High
2_C6_JF_S_1	JF-S-1	Juvenile	F	Vohiparara	VO1	North	High
1_E3_SAF_S_1	SAF-S-1	Subadult	F	Vohiparara	VO3	North	High
4_H5	AM-S-2	Adult	M	Vohiparara	VO2	North	High
4_C5	AF-S-4	Adult	F	Vohiparara	VO4	North	High
4_F1	Y-S-2	Yearling		Vohiparara	VO2	North	High
2_C3_SAF_V_2	SAF-V-2	Subadult	F	Valohoaka	VA2	South	Low
4_E5	JF-T-4	Juvenile	F	Talatakely	T4	South	High
4_D1	AF-S-1	Adult	F	Vohiparara	VO1	North	High
2_G2_AF_S_1	JM-V-2	Juvenile	M	Valohoaka	VA2	South	Low
3_E5	GCGT-T-4	Adult	F	Talatakely	T4	South	High
1_E2_SAM_S_1	AF-S-2	Adult	F	Vohiparara	VO2	North	High
3_H1	SAF-T-1	Subadult	F	Talatakely	T1	South	High
3_F3	SAF-T-1	Subadult	F	Talatakely	T1	South	High
1_B1_PO_T_4	AF-S-1	Adult	F	Vohiparara	VO1	North	High
2_C5_RS_V_2	AM-V-2	Adult	M	Valohoaka	VA1	South	Low
1_E4_SAF_S_1	BGMS-V-1	Adult	F	Valohoaka	VA1	South	Low
3_B2_AM_S_1	PO-T-4	Adult	M	Talatakely	T4	South	High
1_G1_SAM_T_1	AFM-S-2	Adult	F	Vohiparara	VO2	North	High
4_D5	YO-S-4	Adult	F	Vohiparara	VO4	North	High
2_C3_OB_T_1	JF-S-1	Juvenile	F	Vohiparara	VO1	North	High
3_G5	MT-M-1	Adult	M	Mangevo	M1	South	Low
1_H4_JF_S_1	SAF-V-2	Subadult	F	Valohoaka	VA2	South	Low
3_G3	YO-S-4	Adult	F	Vohiparara	VO4	North	High
2_C6_JF_S_1	RS-V-2	Adult	F	Valohoaka	T4	South	Low
1_E3_SAF_S_1	SAM-S-1	Subadult	M	Vohiparara	VO1	North	High
4_H5	RJ-V-1	Adult	M	Valohoaka	VA1	South	Low
4_C5	JM-V-2	Juvenile	M	Valohoaka	VA2	South	Low
4_F1	RS-V-2	Adult	F	Valohoaka	VA2	South	Low
2_C3_SAF_V_2	Y-S-2	Yearling	N/A	Vohiparara	VO2	North	High
2_C6_JF_S_1	SAM-T-1	Subadult	F	Talatakely	T4	South	High
1_E3_SAF_S_1	SAM-S-1	Subadult	M	Vohiparara	VO1	North	High
4_H5	OB-T-1	Adult	M	Talatakely	T1	South	High
4_C5	SAM-V-1	Subadult	M	Valohoaka	VA1	South	Low
4_F1	SAM-V-1	Subadult	M	Valohoaka	VA1	South	Low
2_C3_SAF_V_2	JM-V-2	Juvenile	M	Valohoaka	VA2	South	Low

Table S2: Random replicates ANOVA tests of alpha diversity.

Alpha diversity metric	Replicate #	Variable	F-value	P-value
Shannon's Index	1	Group	2.493	0.0621
		Orientation to the river	2.118	0.1628
		Site	4.217	0.0548
		History of disturbance	1.807	0.1956
		Life history stage	0.657	0.5303
		Sex	2.118	0.1628
	2	Group	2.208	0.0961
		Orientation to the river	0.141	0.7114
		Site	2.823	0.1093
		History of disturbance	5.800	0.0263*
		Life history stage	0.684	0.5168
		Sex	0.684	0.4185
	3	Group	1.750	0.172
		Orientation to the river	0.531	0.475
		Site	0.003	0.9600
		History of disturbance	5.220	0.034*
		Life history stage	0.010	0.9900
		Sex	0.895	0.3560
	4	Group	1.091	0.4050
		Orientation to the river	0.003	0.9550
		Site	0.972	0.3370
		History of disturbance	0.029	0.8660
		Life history stage	0.401	0.6760
		Sex	0.356	0.5580
	5	Group	1.833	0.1490
		Orientation to the river	0.347	0.5630
		Site	0.709	0.4110
		History of disturbance	0.040	0.8440
		Life history stage	0.268	0.7680
		Sex	3.493	0.0780
	6	Group	0.742	0.6020
		Orientation to the river	0.60	0.8090
		Site	0.010	0.9230
		History of disturbance	2.238	0.151
		Life history stage	0.053	0.9490
		Sex	0.007	0.9320
	7	Group	0.832	0.5431
		Orientation to the river	0.834	0.3725
		Site	0.015	0.9037
		History of disturbance	4.245	0.0533
		Life history stage	0.067	0.9355
		Sex	0.560	0.4633
	8	Group	1.978	0.1230

		Orientation to the river	0.011	0.9180
		Site	1.623	0.2190
		History of disturbance	0.488	0.4940
		Life history stage	0.764	0.4810
		Sex	0.859	0.3660
	9	Group	2.115	0.1079
		Orientation to the river	0.165	0.6891
		Site	0.115	0.7379
		History of disturbance	4.815	0.0408*
		Life history stage	0.508	0.6096
		Sex	0.190	0.6680
	10	Group	0.161	0.9738
		Orientation to the river	0.811	0.3792
		Site	0.255	0.6196
		History of disturbance	3.486	0.0774
		Life history stage	0.586	0.5662
		Sex	0.056	0.8150
Faith's PD	1	Group	1.714	0.1750
		Orientation to the river	0.070	0.7940
		Site	1.059	0.3170
		History of disturbance	2.095	0.1650
		Life history stage	1.013	0.3830
		Sex	1.880	0.1870
	2	Group	1.644	0.1968
		Orientation to the river	0.269	0.6097
		Site	0.481	0.4963
		History of disturbance	7.344	0.0139*
		Life history stage	1.000	0.3866
		Sex	2.209	0.1536
	3	Group	1.728	0.1765
		Orientation to the river	0.968	0.3376
		Site	2.238	0.1511
		History of disturbance	11.738	0.0028*
		Life history stage	0.975	0.3954
		Sex	0.174	0.6812
	4	Group	2.212	0.0896
		Orientation to the river	0.407	0.5313
		Site	0.235	0.6336
		History of disturbance	1.137	0.3003
		Life history stage	2.029	0.1604
		Sex	0.995	0.3317
	5	Group	1.471	0.2434
		Orientation to the river	0.639	0.4344
		Site	0.518	0.4809
		History of disturbance	0.015	0.9028

		Life history stage	0.132	0.8776
		Sex	8.398	0.0096*
	6	Group	1.656	0.194
		Orientation to the river	0.064	0.8030
		Site	0.005	0.9440
		History of disturbance	1.059	0.3160
		Life history stage	0.508	0.6100
		Sex	0.448	0.5110
	7	Group	1.367	0.2804
		Orientation to the river	0.232	0.6357
		Site	0.373	0.5487
		History of disturbance	5.059	0.366*
		Life history stage	0.007	0.9931
		Sex	0.232	0.6357
	8	Group	1.123	0.3880
		Orientation to the river	0.159	0.6950
		Site	0.055	0.8170
		History of disturbance	0.285	0.6000
		Life history stage	2.287	0.1300
		Sex	0.001	0.9770
	9	Group	5.017	0.004*
		Orientation to the river	0.106	0.7480
		Site	1.824	0.1927
		History of disturbance	2.914	0.1041
		Life history stage	0.581	0.5689
		Sex	0.343	0.5651
	10	Group	0.670	0.6507
		Orientation to the river	0.472	0.5004
		Site	0.004	0.9590
		History of disturbance	4.983	0.0378*
		Life history stage	1.405	0.2697
		Sex	0.473	0.5001
Observed OTUs	1	Group	1.319	0.2990
		Orientation to the river	0.289	0.5980
		Site	0.947	0.3430
		History of disturbance	0.220	0.6450
		Life history stage	0.941	0.4080
		Sex	2.849	0.1090
	2	Group	1.571	0.2159
		Orientation to the river	0.045	0.8348
		Site	0.674	0.4220
		History of disturbance	6.793	0.0174*
		Life history stage	1.011	0.3825
		Sex	1.982	0.1753
	3	Group	2.367	0.0788

		Orientation to the river	3.057	0.0965
		Site	1.249	0.2777
		History of disturbance	6.931	0.0164*
		Life history stage	0.393	0.6805
		Sex	0.011	0.9189
	4	Group	2.715	0.0467*
		Orientation to the river	0.832	0.3737
		Site	0.358	0.5572
		History of disturbance	0.086	0.7727
		Life history stage	1.831	0.1889
		Sex	0.904	0.3544
	5	Group	0.732	0.6304
		Orientation to the river	0.336	0.5695
		Site	1.267	0.2751
		History of disturbance	0.933	0.3469
		Life history stage	0.483	0.6249
		Sex	3.354	0.0837
	6	Group	1.357	0.2840
		Orientation to the river	0.625	0.4390
		Site	0.013	0.9090
		History of disturbance	1.156	0.2960
		Life history stage	0.491	0.6200
		Sex	0.078	0.7830
	7	Group	0.958	0.4675
		Orientation to the river	0.000	0.9930
		Site	0.019	0.8911
		History of disturbance	4.203	0.0544
		Life history stage	0.016	0.9840
		Sex	0.004	0.9506
	8	Group	0.969	0.473
		Orientation to the river	0.002	0.9690
		Site	0.348	0.5620
		History of disturbance	0.006	0.9380
		Life history stage	1.383	0.2760
		Sex	0.017	0.8990
	9	Group	2.134	0.1054
		Orientation to the river	0.230	0.6371
		Site	1.187	0.2895
		History of disturbance	3.368	0.0822
		Life history stage	0.151	0.8606
		Sex	0.867	0.3634
	10	Group	1.068	0.4089
		Orientation to the river	2.687	0.1176
		Site	0.198	0.6612
		History of disturbance	3.483	0.0775

		Life history stage	1.511	0.2461
		Sex	0.001	0.9759

Table S3: Random replicates Adonis test of beta diversity.

**Beta diversity metric**	**Replicate #**	**Variable**	**R^2**	**P-value**
Bray-Curtis	1	Group	0.32	0.001*
		Orientation to the river	0.17	0.001*
		Site	0.08	0.008*
		History of disturbance	0.05	0.042*
		Life history stage	0.07	0.057
		Sex	0.03	0.139
	2	Group	0.26	0.001*
		Orientation to the river	0.21	0.001*
		Site	0.08	0.003*
		History of disturbance	0.11	0.001*
		Life history stage	0.05	0.087
		Sex	0.02	0.247
	3	Group	0.28	0.001*
		Orientation to the river	0.22	0.001*
		Site	0.07	0.001*
		History of disturbance	0.12	0.001*
		Life history stage	0.06	0.04*
		Sex	0.02	0.178
	4	Group	0.30	0.001*
		Orientation to the river	0.20	0.001*
		Site	0.08	0.004*
		History of disturbance	0.08	0.004*
		Life history stage	0.08	0.024*
		Sex	0.02	0.222
	5	Group	0.20	0.029*
		Orientation to the river	0.22	0.001*
		Site	0.08	0.006*
		History of disturbance	0.12	0.001*
		Life history stage	0.05	0.185
		Sex	0.02	0.258
	6	Group	0.28	0.001*
		Orientation to the river	0.22	0.001*
		Site	0.07	0.008*
		History of disturbance	0.12	0.001*
		Life history stage	0.06	0.027*
		Sex	0.01	0.365
	7	Group	0.31	0.001*
		Orientation to the river	0.20	0.001*
		Site	0.09	0.001*

		History of disturbance	0.08	0.002*
		Life history stage	0.06	0.031*
		Sex	0.02	0.268
	8	Group	0.29	0.001*
		Orientation to the river	0.22	0.001*
		Site	0.08	0.001*
		History of disturbance	0.11	0.001*
		Life history stage	0.05	0.067
		Sex	0.02	0.146
	9	Group	0.34	0.001*
		Orientation to the river	0.20	0.001*
		Site	0.07	0.003*
		History of disturbance	0.08	0.003*
		Life history stage	0.06	0.028*
		Sex	0.02	0.125
	10	Group	0.19	0.025*
		Orientation to the river	0.18	0.001*
		Site	0.08	0.011*
		History of disturbance	0.07	0.023*
		Life history stage	0.08	0.061
		Sex	0.03	0.202
Jaccard	1	Group	0.24	0.001*
		Orientation to the river	0.09	0.001*
		Site	0.05	0.004*
		History of disturbance	0.05	0.007*
		Life history stage	0.06	0.134
		Sex	0.03	0.253
	2	Group	0.23	0.001*
		Orientation to the river	0.11	0.001*
		Site	0.05	0.003*
		History of disturbance	0.06	0.001*
		Life history stage	0.06	0.083
		Sex	0.03	0.155
	3	Group	0.24	0.001*
		Orientation to the river	0.11	0.001*
		Site	0.05	0.001*
		History of disturbance	0.07	0.001*
		Life history stage	0.06	0.07
		Sex	0.03	0.149
	4	Group	0.25	0.001*
		Orientation to the river	0.10	0.001*
		Site	0.05	0.003*
		History of disturbance	0.05	0.004*
		Life history stage	0.06	0.054
		Sex	0.03	0.246

	5	Group	0.23	0.001*
		Orientation to the river	0.11	0.001*
		Site	0.05	0.004*
		History of disturbance	0.06	0.002*
		Life history stage	0.06	0.152
		Sex	0.03	0.134
	6	Group	0.24	0.001*
		Orientation to the river	0.11	0.001*
		Site	0.05	0.003*
		History of disturbance	0.06	0.001*
		Life history stage	0.06	0.040*
		Sex	0.03	0.209
	7	Group	0.25	0.001*
		Orientation to the river	0.10	0.001*
		Site	0.07	0.001*
		History of disturbance	0.06	0.001*
		Life history stage	0.06	0.034*
		Sex	0.03	0.311
	8	Group	0.25	0.01*
		Orientation to the river	0.11	0.001*
		Site	0.04	0.006*
		History of disturbance	0.06	0.001*
		Life history stage	0.06	0.070
		Sex	0.03	0.273
	9	Group	0.21	0.001*
		Orientation to the river	0.10	0.001*
		Site	0.04	0.014*
		History of disturbance	0.06	0.004*
		Life history stage	0.06	0.130
		Sex	0.03	0.285
	10	Group	0.23	0.001*
		Orientation to the river	0.10	0.001*
		Site	0.06	0.001*
		History of disturbance	0.06	0.001*
		Life history stage	0.07	0.027*
		Sex	0.03	0.207
Unweighted UniFrac	1	Group	0.20	0.178
		Orientation to the river	0.09	0.001*
		Site	0.05	0.028
		History of disturbance	0.04	0.056
		Life history stage	0.06	0.479
		Sex	0.03	0.545
	2	Group	0.20	0.002*
		Orientation to the river	0.11	0.001*
		Site	0.05	0.003*

		History of disturbance	0.06	0.001*
		Life history stage	0.05	0.571
		Sex	0.03	0.473
	3	Group	0.25	0.001*
		Orientation to the river	0.10	0.001*
		Site	0.06	0.002*
		History of disturbance	0.08	0.001*
		Life history stage	0.05	0.303
		Sex	0.03	0.175
	4	Group	0.22	0.004*
		Orientation to the river	0.09	0.001*
		Site	0.05	0.005*
		History of disturbance	0.05	0.027*
		Life history stage	0.07	0.080
		Sex	0.03	0.168
	5	Group	0.21	0.028*
		Orientation to the river	0.09	0.001*
		Site	0.04	0.056
		History of disturbance	0.04	0.028*
		Life history stage	0.05	0.555
		Sex	0.06	0.001*
	6	Group	0.21	0.001*
		Orientation to the river	0.10	0.001*
		Site	0.05	0.003*
		History of disturbance	0.04	0.015*
		Life history stage	0.07	0.067
		Sex	0.03	0.279
	7	Group	0.22	0.001*
		Orientation to the river	0.11	0.001*
		Site	0.07	0.001*
		History of disturbance	0.06	0.001*
		Life history stage	0.05	0.374
		Sex	0.03	0.194
	8	Group	0.25	0.002*
		Orientation to the river	0.09	0.001*
		Site	0.05	0.020*
		History of disturbance	0.04	0.018*
		Life history stage	0.06	0.201
		Sex	0.03	0.249
	9	Group	0.19	0.009*
		Orientation to the river	0.09	0.001*
		Site	0.05	0.012*
		History of disturbance	0.04	0.058
		Life history stage	0.06	0.339
		Sex	0.02	0.748

	10	Group	0.20	0.001*
		Orientation to the river	0.12	0.001*
		Site	0.06	0.003*
		History of disturbance	0.05	0.006*
		Life history stage	0.06	0.161
		Sex	0.03	0.385
Weighted UniFrac	1	Group	0.31	0.007*
		Orientation to the river	0.09	0.013*
		Site	0.09	0.013*
		History of disturbance	0.04	0.127
		Life history stage	0.05	0.341
		Sex	0.03	0.294
	2	Group	0.21	0.097
		Orientation to the river	0.07	0.035*
		Site	0.07	0.095
		History of disturbance	0.04	0.255
		Life history stage	0.05	0.410
		Sex	0.02	0.523
	3	Group	0.25	0.015*
		Orientation to the river	0.07	0.033*
		Site	0.04	0.174
		History of disturbance	0.12	0.006*
		Life history stage	0.05	0.402
		Sex	0.03	0.325
	4	Group	0.29	0.010*
		Orientation to the river	0.08	0.015*
		Site	0.08	0.016*
		History of disturbance	0.02	0.400
		Life history stage	0.09	0.073
		Sex	0.02	0.557
	5	Group	0.34	0.014*
		Orientation to the river	0.08	0.033*
		Site	0.06	0.061
		History of disturbance	0.03	0.343
		Life history stage	0.02	0.870
		Sex	0.02	0.625
	6	Group	0.23	0.047*
		Orientation to the river	0.08	0.025*
		Site	0.04	0.190
		History of disturbance	0.12	0.005*
		Life history stage	0.03	0.723
		Sex	0.02	0.637
	7	Group	0.17	0.340
		Orientation to the river	0.09	0.036*
		Site	0.03	0.406

		History of disturbance	0.05	0.184
		Life history stage	0.04	0.667
		Sex	0.04	0.237
	8	Group	0.44	0.001*
		Orientation to the river	0.05	0.054
		Site	0.07	0.022*
		History of disturbance	0.04	0.085
		Life history stage	0.04	0.320
		Sex	0.04	0.102
	9	Group	0.35	0.001*
		Orientation to the river	0.08	0.008*
		Site	0.05	0.051
		History of disturbance	0.09	0.006*
		Life history stage	0.08	0.063
		Sex	0.01	0.912
	10	Group	0.16	0.432
		Orientation to the river	0.05	0.218
		Site	0.04	0.221
		History of disturbance	0.05	0.204
		Life history stage	0.07	0.296
		Sex	0.02	0.637

Table S4: No clear effect of geographic distance on gut microbiome beta diversity. Results of pairwise PERMANOVA tests of beta diversity for the independent variable *sampling site* using the mean-ceiling dataset. Sites are arranged from north to south in the header row and column (see Fig. 1 and Table 1). To show the magnitude of microbiome difference between sites, cells denote *pseudo-F* values. Asterix indicate significant q-values (q < 0.05). The top half of the matrix (shaded light orange) shows Jaccard values; the bottom half (shaded light gray) shows Bray-Curtis. For both metrics, the magnitude of differentiation does not increase with distance (i.e., Vohipara and Mangevo are not the most different).

**Site**	**Vohiparara**	**Talatakely**	**Valohoaka**	**Mangevo**
**Vohiparara**	x	4.17*	4.49*	2.98*
**Talatakely**	22.76*	x	2.45*	3.10*
**Valohoaka**	9.41*	2.97	x	2.62*
**Mangevo**	3.78*	12.99*	3.73	x
